# Photolytic Mass Loss of Secondary Organic Aerosol Derived from Photooxidation of Biomass Burning Furan Precursors

**DOI:** 10.1021/acsestair.4c00230

**Published:** 2025-03-11

**Authors:** Nara Shin, Bin Bai, Taekyu Joo, Yuchen Wang, Nga L. Ng, Pengfei Liu

**Affiliations:** † School of Earth and Atmospheric Sciences, Georgia Institute of Technology, Atlanta, Georgia 30332, United States; ‡ Department of Earth and Environmental Sciences, Korea University, Seoul 02841, South Korea; § School of Chemical and Biomolecular Engineering, Georgia Institute of Technology, Atlanta, Georgia 30332, United States; ∥ College of Environmental Science and Engineering, Hunan University, Changsha, Hunan 410082, China; ⊥ School of Civil and Environmental Engineering, Georgia Institute of Technology, Atlanta, Georgia 30332, United States

**Keywords:** biomass burning, secondary organic aerosol, photolysis, brown carbon, elemental composition, furan

## Abstract

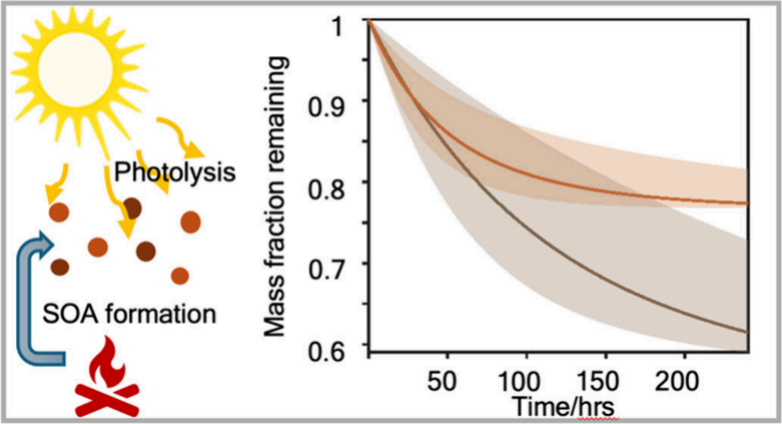

Direct photolysis as a potentially important chemical loss pathway for atmospheric organic aerosol (OA) is increasingly recognized but remains highly uncertain, particularly for secondary organic aerosol (SOA) derived from biomass burning (BB) precursors. We present the measurements of the photolytic mass change of SOA derived from photooxidation of three furan precursors, 3-methylfuran, 2-methylfuran, and furfural, in an environmental chamber under both dry and humid conditions. Each type of SOA was collected on crystal sensors, and the mass losses by photolysis under 300 or 340 nm light were continuously monitored using a quartz crystal microbalance (QCM). By incorporation of measurements and modeling, 10–40% of furan SOA masses can be lost by direct photolysis under solar radiation over their typical atmospheric lifetime. The mass loss fraction is well correlated with the mass fraction of nitrogen-containing compounds (NOC) in the SOA, possibly because these species can largely enhance the light absorption cross section and readily undergo photodissociation under UV light.

## Abstract 2

Biomass burning is a large source of volatile organic compounds, such as furan species, which further produce secondary organic aerosol (SOA) in the atmosphere. The measurements and modeling suggest that 10−40% of the furan SOA masses can be photolyzed under solar radiation over its typical atmospheric lifetime.

## 1 Introduction

Biomass burning emits large amounts of primary organic aerosol (POA) as well as volatile organic compounds (VOCs) as products of cellulose pyrolysis.^1−3^ The emitted VOCs can be oxidized in the atmosphere to produce secondary organic aerosol (SOA).^4,5^ Those directly emitted (primary) and chemically produced biomass burning organic aerosols (BBOAs) can further undergo various chemical and physical aging processes, such as condensational growth, evaporation, coagulation, chemical oxidation, hydrolysis, and photolysis, which can change their climate- and health-relevant properties.^6−13^ Recent studies have identified that aerosol and oxidation products of VOCs emitted by biomass burning can change the radiative forcing in the range from −0.8 to 0.6 W/m^2^ on the climate system, introducing a large uncertainty into the assessment of historic and future climate change.^14−16^

While many VOCs are emitted into the atmosphere in the event of biomass burning, furan species have been confirmed as dominant precursors of biomass burning SOA.^17^ A significant yield of SOA along with other oxidation products has been reported for furan species in previous laboratory studies,^18−21^ and the abundance of gaseous furan species and their SOA formation potential have been quantified in a few field studies.^17,22,23^ More specifically, the oxidation of furan species by the hydroxyl radical (OH) shows a SOA yield of 1.85–8.5% for a furan SOA mass concentration of ≤68.7 μg/m^3^, along with carbonyl formation from chemical analysis.^19^ Laboratory results from furan species oxidized by nitrate radical (NO_3_) show a 1.6–2.4% SOA yield for a generated aerosol mass concentration of 5–45 μg/m^3^, contributing to the nighttime formation of SOA.^18,20−22^ Coggon et al.^22^ evaluated furan species as one of contributors of ozone and maleic anhydride formation by combining FIREX-AQ measurement with the master chemical mechanism (MCM) model.

Along with SOA formation pathways, the change in the chemical and physical properties of SOA continues during the atmospheric transport processes, including multiphase/heterogeneous reaction due to photochemical aging.^24^ Although multiphase/heterogeneous oxidation of SOA particles by gaseous oxidants, such as OH radical, is traditionally considered the most important pathway of photochemical aging, recent studies highlight that in-particle reactions directly induced by UV can also initiate SOA aging.^25−28^ One mechanism involved in photolytic aging is photosensitization, which contributes to aqueous formation of SOA and brown carbon and to the transformation of optical properties, such as photobleaching and photoenhancement.^29−34^ A few chamber studies have investigated the impact of photolysis on SOA mass, showing the reduction of SOA mass in the presence of UV light at low OH concentrations, in which photolysis could have contributed substantially to SOA volatilization and mass loss.^25,35,36^ Modeling studies suggest that including the photolysis effect as an atmospheric SOA sink (in addition to wet and dry deposition) can strongly reduce the atmospheric lifetime and alter the vertical distribution of SOA, suggesting a more dynamic picture of the SOA life cycle.^37,38^

Despite its importance in atmospheric processes, there has been a limited number of studies performed to determine the impact of direct photolysis on SOA. Some studies have noted limitations in using aircraft measurement to assess changes in BBOA mass during plume evolution, as the mass enhancement from SOA formation can be offset by volatilization due to dilution, oxidation, and photolysis.^39,40^ Additionally, spatial and temporal variability in fire emissions and non-Lagrangian nature of aircraft trajectories add complexity to data interpretation.^41^ These factors contribute to uncertainties in identifying the impact of photolysis on BBOA in field studies, underscoring the importance of laboratory studies. In chamber experiments, it remains challenging to separate direct photolysis from other processes, such as OH oxidation, particle and vapor wall losses, and the temperature effect induced by UV light. Typical chamber experiments are conducted on a time scale of less than 1 day, which is shorter than the atmospheric lifetime of SOA.^39,40^ To overcome these limitations, a few previous studies have utilized the quartz crystal microbalance (QCM) to characterize the mass changes of collected SOA films induced by direct UV photolysis.^42−44^ This technique enables long measurements over a time scale relevant to the SOA lifetime (days to 1 week) in a controlled gas environment, which is highly valuable for the photolysis measurements. The QCM technique has also been applied in previous studies to measure the volatility, diffusivity, and hygroscopicity of SOA.^45−47^ Nevertheless, further understanding of the atmospheric process pathways (i.e., photolysis) of BBSOA is still required to fully understand its impact on climate. In particular, the implication of direct photolysis on BBSOA has not been studied in the laboratory, and key parameters needed to estimate the SOA fate in three-dimensional chemical transport models are still unknown.

In this study, we produced SOA from photooxidation of different furan species that are prevalent in biomass burning emissions, including 3-methylfuran, 2-methylfuran, and furfural, in an environmental chamber under both dry and humid conditions and measured the chemical composition of SOA using a high-resolution time-of-flight aerosol mass spectrometer (HR-ToF-AMS). We grew thin films of the SOA material by collecting the produced SOA on crystal sensors. The mass changes of SOA film during direct photolysis under 300 and 340 nm UV lights were continuously monitored using a high-sensitivity, temperature-controlled QCM with dissipation (QCM-D) apparatus. We then correlated the chemical characteristics measured by the HR-ToF-AMS to investigate specific aerosol components contributing to the photodegradation process. Based on the two-wavelength measurements, we further estimated the photolytically induced mass change of each SOA type under the solar spectrum, and the atmospheric implications of photolysis of BBSOA as an OA loss mechanism are discussed.

## 2 Materials and Methods

### 2.1 Laboratory Chamber Experiment: SOA Generation

All experiments for SOA production were performed in the 12 m^3^ Georgia Tech Environmental Chamber (GTEC) facility equipped with multiple instruments, which simultaneously measure the aerosol mass concentration, the chemical composition of the formed SOA, the levels of NO, NO_2_, and O_3_, the relative humidity (RH), and the temperature.^48^ Wet ammonium sulfate seed particles produced by atomizing a 0.015 M (NH_4_)_2_SO_4_ solution (model 3076, TSI) were injected into the chamber without drying. The chamber was operated under two different relative humidity conditions (<5% and ∼50% RH), such that the ammonium sulfate particles adopt solid and liquid phase states, respectively.

For the production of SOA, three different types of biomass burning VOC precursors, 3-methylfuran (98%, Acros Organics), 2-methylfuran (99%, Sigma-Aldrich), and furfural (99%, Sigma-Aldrich), were chosen owing to their prevalence in biomass burning smoke and SOA formation potential.^19−21^ Nitrous acid (HONO) was used as a source of the OH radical in this study. HONO was generated by a liquid phase reaction of 10% sodium nitrite (NaNO_2_, VWR International) with 10% diluted sulfuric acid (H_2_SO_4_, VWR International). The mixture of two liquids formed gas phase HONO, which was transferred in a stream of pure air at a flow rate of 5 L/min.^49^ After seed particle injection, a VOC precursor was injected into the chamber with HONO. Detailed information about chamber experiments can be found in Table S1. Briefly, the reacted VOC concentrations were 520–530 ppb for 3-methylfuran, 600–615 ppb for 2-methylfuran, and 197–201 ppb for furfural (Table S1), roughly consistent with our previous study.^20^ We used relatively high precursor concentrations in these studies to ensure that sufficient SOA mass can be collected for offline analyses. An excess of HONO was added to the chamber. After 1 h of mixing, the chamber UV lights (300–400 nm) (Sylvania, 24922) were illuminated to generate the OH radical by photolyzing HONO, which subsequently initiated the formation of SOA.

A scanning mobility particle sizer (SMPS, model 3080, TSI) was used to measure the particle number and volume diameter distributions for particles inside the chamber in the diameter range from 17 nm to 1 μm. The concentration of the injected VOC was measured online using a gas chromatograph flame ionization detector (GC-FID, 7890A, Agilent Technologies). The changes in the NO_*x*_ cycle from HONO injection were measured by a chemiluminescence NO_*x*_ monitor for NO (42C, Thermofisher Scientific) and cavity-attenuated phase shift (CAPS) for the NO_2_ concentration. The SOA composition was also monitored by the HR-ToF-AMS (Aerodyne Research, Inc.).^50,51^ HR-ToF-AMS data were analyzed using Igor pro 8.0.4.2 with the standard analysis toolkit of Squirrel version 1.65 and Pika version 1.25. Chemical analysis results presented in this study were obtained by high-resolution peak fitting, and elemental ratios were derived from the improved ambient method.^52^ In each system, chemical characteristics of SOA were obtained by averaging HR-ToF-AMS measurements over a 30 min period at the peak of the organic mass concentration produced in the chamber, which aligned with the sample collection time for the QCM analysis. The retrieved chemical composition data were used for correlation analysis to assess the potential factors that affect photolysis.

### 2.2 Photolysis of Collected SOA Films on a QCM

A high-sensitivity QCM (Q-sense explorer, Biolin Scientific AB; with a limit of detection of 1.34 ng/cm^2^ (Figure S1)) was used to measure changes in SOA mass collected on quartz crystal sensors during UV photolysis.^36^ For each QCM sensor, water and methanol were sequentially used to rinse the surface of the sensor. The rinsed sensors were dried with zero air and then treated with UV–ozone (UV–ozone cleaner, ProCleaner Plus, Bioforce Nanoscience) for 20 min to remove any residual contaminants, including organics and VOCs on the surface. After the UV–ozone treatment, sensors were placed in a QCM for baseline measurements. The baseline was needed to identify the deposited mass on the QCM sensor after SOA collection.

The collection of SOA was conducted using two different methods: (1) a custom-made single-stage impactor^45,53,54^ and (2) a corona-discharge unipolar charger (CC-8020, IONER) combined with a nanometer aerosol sampler (TSI 3089). The custom-made single-stage impactor was connected to the chamber, and the chamber air was directly drawn at a flow rate of 4 L/min. The flow rate allotted here gave a cutoff diameter of 100–200 nm, which was sufficient to collect the produced SOA in the chamber onto QCM sensors located inside the impactor. The other collection method used a nanometer aerosol sampler with a sensor placed on a negative electrode stage (−10 kV) to collect particles positively charged by a unipolar charger. The flow rate was 1 L/min. By using a unipolar charger operated in the positive mode, this combined apparatus can largely increase the particle collection efficiency (∼70–100% for particles smaller than 500 nm) compared with our previous studies using a bipolar charger (∼2–10%) for PM_1_.^45,46,55^ Therefore, both methods can effectively collect SOA produced in the chamber with a mode diameter of ∼300–500 nm in mass–diameter distribution, as measured by the SMPS. To minimize the artifact, samples were collected over a period of 30 min to 1 h. Collected samples were immediately analyzed using a QCM or stored at −20 °C until analysis. The custom-made single-stage impactor with a single nozzle produced a relatively non-uniform pattern of SOA collection with a thicker layer under the nozzle that spread due to particle rebound, whereas the nanometer sampler yielded a more uniform, thin film morphology. Both sampling methods produced roughly consistent results for the photolytic mass loss experiments (Figure S2). The nanometer aerosol sampler method produces SOA films with more uniform particle deposition and lower uncertainty, making it the preferred approach for future QCM experiments. The sample masses and sampling methods used for analyses in this study can be found in Table S1.

The SOA-laden QCM sensor was placed in a window module flow cell (Biolin Scientific, Q-sense QWM-401), and the SOA mass was analyzed under different UV light wavelengths (300 and 340 nm and in the dark) (UV LED M300L4 and M340L4, respectively, Thorlabs) using a QCM as shown in Figure S1. The selected UV wavelengths (300 and 340 nm) represent UV-B and UV-A radiation, respectively, relevant to solar radiation under tropospheric conditions. Using narrow-band LED UV light sources minimizes excess heat generation, which is common with broadband light sources like xenon lamps used in solar simulators. The sampling frequency of QCM was roughly 1 s. The height of the LED light was affixed to be identical throughout the entire experiment to maintain the UV light intensities. The UV photon flux was measured by a power energy meter (PM100D with a S405C sensor, Thorlabs). The QCM chamber was purged with a zero air flow (737-15, Aadco) at a constant flow rate of 0.03 L/min controlled by a mass flow controller (MFC) (Alicat Scientific). The zero air used in this study was dry below 1% RH. The temperature of the QCM flow cell was actively maintained at 20 °C with a long-term stability of <0.02 °C/h using a Peltier thermoelectric module, ensuring stable environmental conditions and minimizing fluctuations in QCM mass measurements. Although the QCM chamber itself reaches the target RH within 1–2 min, an initial 10–20 min of background equilibration time was allotted to allow the samples to adjust fully to the target RH. This equilibration period was excluded from data analysis. The UV lights were alternated on and off every 10 min for a clear distinction between photolysis and nonphotolysis periods. The light-off period between two light-on periods represented evaporation without active direct photolysis, which can be used to determine the semivolatile species of the photolysis products. The effective UV flux reported in this study was half of the measured UV flux, accounting for the on–off cycle of UV light.

The thickness of the collected SOA film, calculated from QCM mass measurements under the assumption of uniform deposition, ranged from 0.2 to 1.36 μm. Absorptive index *k* values of derived SOA, estimated using UV–vis spectrophotometer, ranged from 0.004 to 0.08 at λ = 310 nm.^20^ Penetration depth *δ*_p_ can be calculated as , yielding values between 0.3 and 6 μm. Based on these calculations, weakly absorbing SOA samples were optically thin, allowing uniform UV flux throughout the SOA film. However, for thicker and more strongly absorbing SOA samples, UV irradiation can be attenuated within the film.

The reproducibility and uncertainty of the photolytic mass loss measurements were tested to ensure the reliability of the results, as shown in Figure S2. The repeated measurements demonstrated consistent reproducibility within the experimental uncertainty. Dark experiments without turning on the UV light were also conducted to measure the evaporative mass loss of SOA without photolysis. The data were reported as the mass fraction remaining of SOA, defined as the SOA mass during the dark or photolytic aging experiment normalized by the initial SOA mass deposited on the QCM sensor. Compared to the absolute mass, the metrics of the mass fraction remaining can better account for variations in the deposited mass from sensor to sensor. The analyses of SOA mass fraction remaining have excluded the mass of ammonium sulfate seed particles for each experiment (i.e., assuming ammonium sulfate is not photolabile), determined based on the ammonium sulfate/OA mass ratio from the HR-ToF-AMS analysis for chamber aerosol during the sampling period.

### 2.3 Modeling of SOA Mass Loss by Photolysis under the Solar Spectrum

We developed a simplified model to demonstrate the potential impact of photolysis on SOA under solar irradiation under atmospheric conditions. Details about the model are described in section S1 of the Supporting Information. Briefly, this model specifically utilized experimental results from both UV lamps (with nominal wavelengths of 300 and 340 nm, respectively) and predicted the decay of furan SOA mass under sunlight.

Since not all SOA mass deposited on the sensor undergoes photolysis, we expressed relevant variables obtained from experimentally measured photolytic decay under each lamp using exponential functions. The exponential function can be displayed as in eqs 1 and 2. In these equations, we fitted the nonphotolabile fraction (i.e., less volatile fraction of OA that remains in the particle phase after photolytic reactions; *A*_0_ for 300 nm and *B*_0_ for 340 nm), the photolabile fraction (i.e., vaporized fraction after photolytic reactions; *A*_1_ for 300 nm and *B*_1_ for 340 nm), and the photolysis rate (*k*_1_ for 300 nm and *g*_1_ for 340 nm). We further assumed that a faction of SOA can be photolyzed by both 300 and 340 nm, a fraction can be photolyzed by one light only (typically 300 nm), and a fraction is nonphotolyzable. The sum of these fractions equals unity (see eq S13–S17 of the Supporting Information):
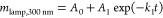
1
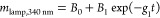
2

In order to investigate the mass decay of furan SOA through photolysis under solar spectrum conditions, we employed scaling factors to convert the photolysis rates under two UV lamps into the rates under two solar UV bands (UV-B and UV-A):
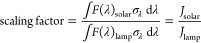
3
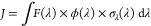
4where *F* is the photon flux and *σ*_*λ*_ is the absorption cross section. The photo fluxes of solar radiation were derived from the standard solar irradiance spectrum (Air Mass 1.5, ASTM G-173-03, National Renewable Energy Laboratory), representing hemispherical solar irradiance (direct and sky diffuse light) at a zenith angle of 48° representative of the contiguous United States.

The ratios between *J*_lamp_ and *J*_solar_ were used to derive scaling factors for each lamp. The implied assumption here is that quantum yield *ϕ*_L,λ_ is constant within the wavelength range of each lamp. The two derived scaling factors for 300 and 340 nm UV lamps were applied to solar UV light from 290 to 330 nm and from 330 to 400 nm, respectively (Figure S3). The uncertainty associated with the cutoff wavelength (330 ± 10 nm) between two wavelength bands has been considered in the error estimates. The wavelength-dependent *σ*_*λ*_ values were measured for SOA methanol extracts by UV–vis spectroscopy (DT-Mini and USB4000, Ocean Optics) (Figure S4). Furthermore, the mass absorption efficiency (MAE) for each compound was determined based on the absorbance measured as described in section S2.

With the application of scaling factors, the mass fraction loss of each experiment is regenerated, equivalent to solar radiation. With the wavelength-dependent absorption cross section, the photolysis rate constant of solar radiation (*J*_solar_) was also calculated (eq 4). More details about the conversion can be found in section S1. This approach allowed us to assess the impact of solar radiation on the degradation of furan SOA utilizing experimental results from two wavelengths, providing valuable insights into its environmental fate and potential implication.

## 3 Results and Discussion

### 3.1 Photolytic Mass Loss of SOA Generated under Dry and Humid Conditions

**Figure 1:**
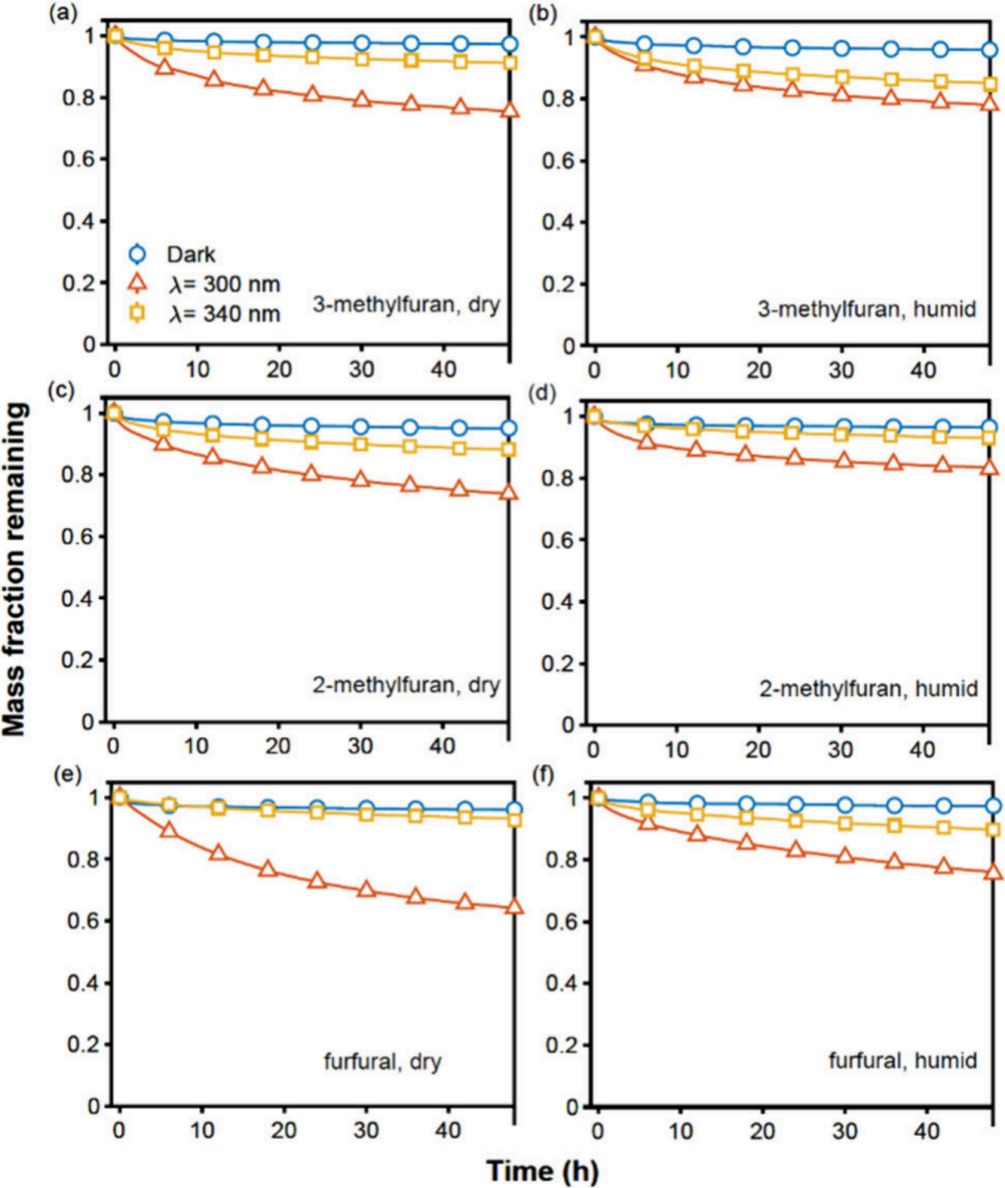
Time series profiles of the SOA mass fraction remaining measured by a QCM for photolysis under 300 nm (red) and 340 nm (orange) UV light irradiation and during dark (blue) aging under dry conditions in the QCM cell: (a) 3-methylfuran SOA generated under the dry chamber condition, (b) 3-methylfuran SOA generated under the humid chamber condition, (c) 2-methylfuran SOA generated under the dry chamber condition, (d) 2-methylfuran SOA generated under the humid chamber condition, (e) furfural SOA generated under the dry chamber condition, and (f) furfural SOA generated under the humid chamber condition. The mass of ammonium sulfate seed particles has been excluded using the HR-ToF-AMS measurements for chamber experiments during sample collection.

A series of experiments confirm that SOA produced from different furan precursors can undergo significant photolytic mass loss under both 300 and 340 nm UV irradiation. Figure 1 shows the measured mass fraction remaining time profiles for SOA generated from three different VOC precursors under different chamber RHs. The reacted precursor concentrations, SOA elemental ratios, and organic mass loadings collected on the QCM sensors for all experiments are listed in Table S1. The O/C ratios for produced SOA range from 0.94 to 1.55, indicating these particles are highly oxidized. The final percentages of SOA mass loss after 48 h under the dark condition and under 300 and 340 nm UV light in the QCM are summarized in Table S2. The mass fraction losses observed under dark conditions (blue line with circular markers) are close to or less than 5% for all types of SOA studied. This mass loss is caused by the evaporation of semivolatile and intermediate-volatility organic compounds (S/I VOCs) that occurred in the QCM flow cell, which is determined by the intrinsic volatility and possible diffusivity of the SOA material.^37^ The mass fraction losses for photolytic aging under 300 and 340 nm UV irradiation are larger than under the dark conditions over the entire 48 h experiments, indicating substantial photolytic mass losses. For SOA produced under dry conditions, the percentage of mass loss after 48 h photolysis under 300 nm UV is the greatest for furfural SOA (35.8%), followed by 2-methylfuran SOA (26.2%) and 3-methylfuran SOA (24.5%). Meanwhile, SOA produced in the humid chamber shows less fraction loss compared to dry SOA (23.9%, 16.5%, and 22.0% for furfural, 2-methylfuran, and 3-methylfuran SOA, respectively). These differences likely arise from variations in the SOA chemical composition. For example, furfural SOA produced under humid conditions exhibits a lower O:C ratio compared to its counterpart produced under dry conditions (Table S1), emphasizing the role of the production conditions in shaping SOA composition. However, elemental ratios alone do not fully account for the observed differences in photolytic mass loss fractions across different SOA types and production conditions, suggesting that additional factors, such as molecular structure, functional group distribution, and light absorption properties, may also play a crucial role (see section 3.2 for further discussion). Compared with photolytic aging experiments under 300 nm UV light for 48 h, the mass loss fractions for the photolysis induced by 340 nm UV light aging for 48 h are smaller. This can be explained by the weaker light absorption and possibly lower quantum yield at the longer wavelength, although the 340 nm UV light used in this study had a 3 times stronger light intensity than the 300 nm light (Figure S2).

These results can be compared with those of previous studies on the photolytic mass loss of different types of SOA measured online in chamber studies, offline by the QCM, and other techniques. For example, chamber studies by O’Brien and Kroll^56^ suggest that α-pinene SOA under 300–400 nm UV light undergoes rapid initial mass decay and 70–90% SOA mass is photorecalcitrant with negligible photolytic degradation. Our study confirms that a large fraction (60–90%) of SOA derived from furan species can be nonphotolabile.

### 3.2 Relationship between Chemical Characteristics of Furan SOA and Mass Fraction Loss

**Figure 2:**
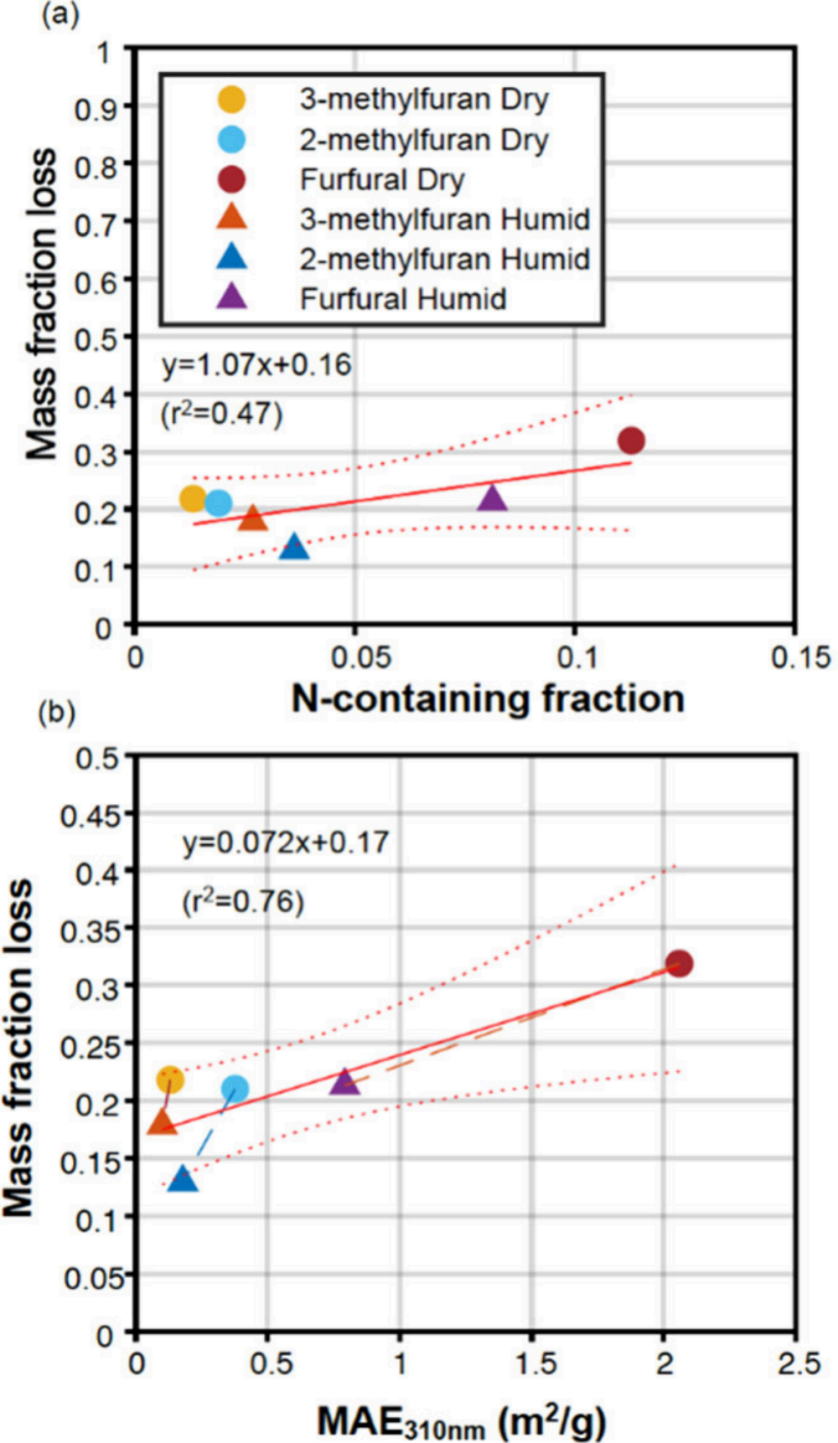
Chemical characteristics vs mass fraction loss after 48 h under 300 nm UV irradiation of furan SOA. Mass fraction loss vs (a) N-containing fraction and (b) MAE at 310 nm measured by a UV–Visible spectrophotometer. The solid line represents the fitted line from linear regression, and the dotted lines represent the 95% confidence interval.

The photolysis behavior of the SOA can be affected by several factors. The actinic flux of UV irradiation and the light absorption cross section of SOA combined can determine the photon flux absorbed by the SOA, which can in turn determine the upper limit of photolysis rates (eq 4). The chemical composition, such as functional groups, can determine both light absorption and the quantum yield, thus influencing the photolysis rates and photolabile fraction. The mass loss rate and mass remaining fraction can also be influenced by the volatility of photolysis products as photolysis may produce S/I VOCs that slowly evaporate even without UV light. Using SOA chemical composition data measured by the HR-ToF-AMS, we explored potential relationships between the mass loss fraction and the mass fraction of different chemical families, including nitrogen-containing organic compounds (NOCs), the C*_x_*H*_y_*O family with only one oxygen (CHO_1_), the C*_x_*H*_y_*O*_z_* family with more than one oxygen (CHOGT_1_), the C*_x_*H*_y_* family, and the C*_x_* family (Figure S5). The SOA composition measured by the HR-ToF-AMS for each type of experiment is shown in Figure S5. Due to the weak light absorption of organic nitrates (NO family), we only included the CHN (reduced nitrogen-containing species without an oxygen atom), CH_(*y*)_ON (nitrogen-containing species with only one oxygen atom), and CHO_(*y*)_N (nitrogen-containing species with more than one oxygen atom) families as nitrogen-containing organic compounds (NOCs) in this analysis.^20,57,58^ This nitrogen-containing fraction may contain a carbonyl group that could potentially initiate photosensitizing reactions under the solar spectrum. Among the measured chemical families, the nitrogen-containing fraction demonstrated the strongest correlation with the photolabile fraction, while others showed weaker associations. Figure 2a shows the relationship between the mass fraction loss obtained under 300 nm after a 48 h UV light irradiation and the fraction of NOCs, which is the chemical family showing the strongest correlation with mass fraction loss for 300 nm UV (slope = 1.07, and *r*^2^ = 0.47). We further correlated mass fraction loss with MAE at 310 nm. MAE_310 nm_ was used due to the uncertainties of methanol soluble extracts at wavelengths below 305 nm on the UV–vis spectrometer. As shown in Figure 2b, a higher MAE_310 nm_ is associated with a larger mass fraction loss for 300 nm UV photolysis with a slope of 0.07 and an *r*^2^ of 0.76.

The chemical composition of the furan SOA is expected to impact the photolysis and mass fraction loss observed in the QCM experiment. Via detailed analysis of the bulk aerosol composition, light absorption of furan SOA produced under different chamber RHs and possible chemical mechanisms of brown carbon formation have been discussed in our previous study by Joo et al.^20^ NOC is an important contributor to light absorption. Specifically, it has been shown that NOCs, dominated by amines and amides produced from reactions between carbonyls and ammonia/ammonium, are associated with brown carbon formation in the photooxidation of furan compounds.^20^ Here, Figure 2 indicates that furfural SOA having a higher light absorption properties at 310 nm is associated with the larger mass fraction loss by photolysis. This result suggests that NOCs might affect photolysis by serving as chromophores, contributing significantly to light absorption at 310 nm and being susceptible to loss through photobleaching.^56,58,59^ In particular, the difference in light absorption properties between SOA produced under dry and humid chamber conditions of the same precursor can explain the difference in the photolabile fraction under 300 nm UV light. Besides, a weaker correlation between the mass loss fraction at 340 nm and MAE_340 nm_ was found (*r*^2^ = 0.28) compared with results for 300 nm UV (*r*^2^ = 0.76), which might result from the nonlinear absorption behavior of SOA brown carbon species. For example, a minor group of species that absorb at 340 nm can contribute to the light absorption, but the mass could be relatively small. As a result, there will be only minor photolytic mass depletion. Another plausible mechanism is that photolysis at 340 nm UV is mostly limited by the quantum yield rather than the absorption coefficient.

We noticed that the mass fraction of NOCs (1.3–11.3%) is smaller than the mass fraction loss (13–32% for 48 h exposure under 300 nm UV), indicating that photolysis of nitrogen-containing species alone does not fully explain the photolabile fraction of SOA. Therefore, we also explored the relationships between mass fraction loss and other species. In previous research, Wong et al.^60^ utilized the analysis method adopted from Ng et al. in 2011^61^ to describe the trend of the main fragments measured by the HR-ToF-AMS and identified a substantial decrease in the fraction (i.e., fraction of C_2_H_3_O^+^, mainly fragments from carbonyls, among other species such as alcohols and ethers) during the photolysis period from α-pinene SOA, as carbonyls can be chromophores that are photolyzable. In this study, we examined the relationship of the photolytic mass fraction loss at 300 nm with *f*_43_ for different fresh SOA types, but the results did not show a significant relationship (Figure S6). This analysis was based on the bulk initial chemical composition of the SOA as measured by the HR-ToF-AMS. However, carbonyl groups, particularly those associated with aromatic ring structures, may serve as photosensitizers during photolytic aging. Photosensitization involves the formation of excited triplet states of organic compounds upon solar irradiation followed by a new generation of reactive radicals and oxidation of less reactive compounds. Photosensitizers, including aromatic carbonyls, might be present in the investigated SOA and potentially contribute to the overall photolysis reactions.^29−34^

As a caveat, as our study only measured the chemical composition of fresh SOA before photolysis, the poor correlation could be a result of large variations in the initial *f*_43_ across different SOA types. Future studies need to investigate the changes in chemical composition of the SOA during and after photolysis to identify the relationship between the photolytic mass loss and the changes of aerosol properties. Another plausible explanation is that the functional groups that dominate photolysis behaviors can differ depending on the major chemical oxidation pathways (i.e., ozonolysis in ref (60) versus photooxidation in the presence of NO_*x*_ used in this work). In addition, the C_2_H_3_O^+^ fragment measured by the HR-ToF-AMS does not directly identify functional groups or molecular chemical compositions. The oxidation of different precursors can produce different species even though these species could have similar fragments. These results underscore the need for future studies on how the molecular composition of SOA can influence its photolability.

### 3.3 Atmospheric Implications

**Figure 3:**
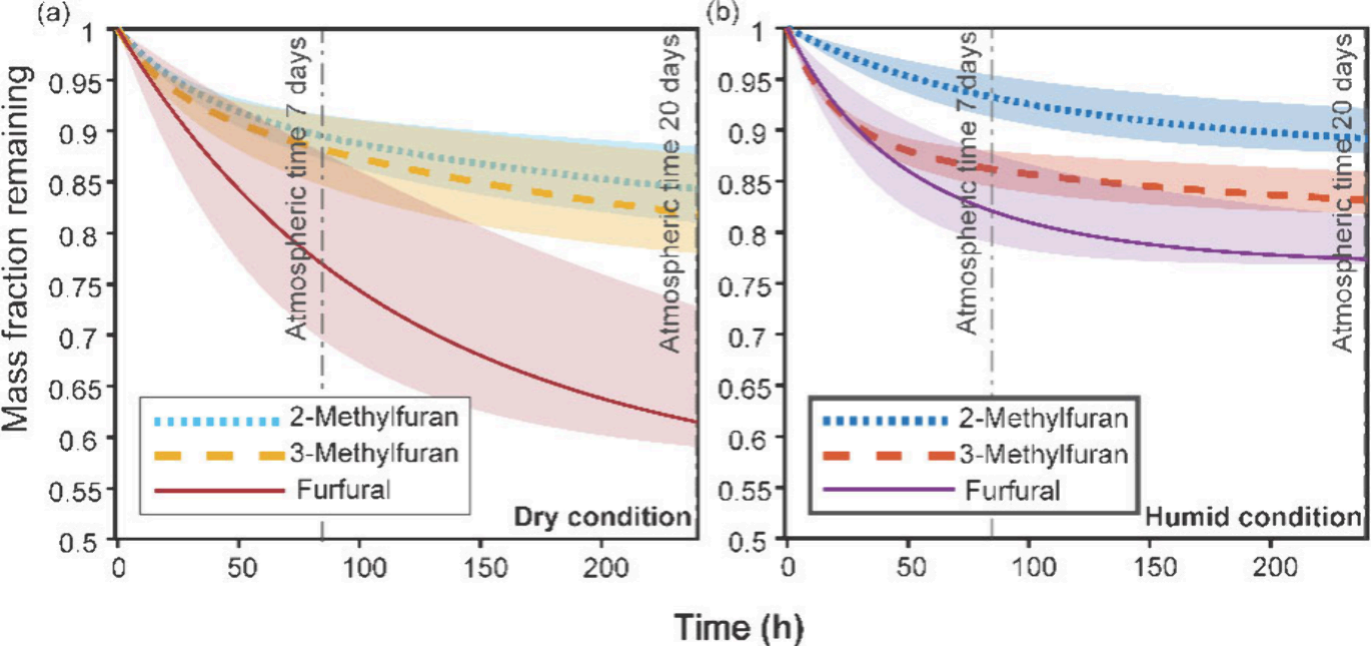
Photolytic mass loss curves modeled under solar spectrum radiation for each SOA type: (a) photolytic decay of SOA formed in the chamber under dry conditions and (b) photolytic decay of SOA formed in the chamber under humid conditions. Vertical lines represent the atmospheric equivalent time of photolysis. The shaded area represents the error ranges of the mass fraction remaining.

The nonphotolabile fraction (*A*_0_ for 300 nm and *B*_0_ for 340 nm), photolabile fraction (*A*_1_ for 300 nm and *B*_1_ for 340 nm), and photolysis rate (*k*_1_ for 300 nm and *g*_1_ for 340 nm (see eqs 1 and 2)) were determined by exponential fitting based on the experiments under monochromatic light. These results indicate that only a fraction of SOA mass is photolabile (i.e., *A*_1_ and *B*_1_ values in Table 1), and the lifetime of this fraction is typically tens of hours under laboratory UV light conditions. To better understand the mass decay of SOA under sunlight, we used a biexponential fitting to obtain the mass loss under the solar spectrum as described in Materials and Methods and section S1. We projected photolysis to atmospheric conditions; the derived photolysis curves are shown in Figure 3, and the potential mass losses after photolysis for 24, 48, 72, 84, 120, and 240 h are listed in Table S3. A smaller mass loss was derived under ambient conditions compared to the experimental results at the same photolysis time, because of the differences in light intensity. Among the examined SOA types, the largest estimated potential mass fraction loss was observed for furfural SOA produced under dry conditions, which is a strong brown carbon.^20^

**Table 1:** Photolabile, Nonphotolabile, and Mass Decay Rate for Each SOA Type

	SOA precursor	*A*_0_	*A*_1_	*k*_1_ (h^–1^)	*B*_0_	*B*_1_	*g*_1_ (h^–1^)
dry	3-methylfuran	0.76	0.24	0.057	0.94	0.061	0.074
	2-methylfuran	0.77	0.23	0.058	0.93	0.071	0.064
	furfural	0.62	0.38	0.043	0.27	0.73	0.00088
humid	3-methylfuran	0.81	0.19	0.068	0.89	0.11	0.082
	2-methylfuran	0.87	0.13	0.087	0.95	0.049	0.026
	furfural	0.77	0.23	0.049	0.92	0.083	0.046

The dashed vertical lines mark the potential decay in the mass fraction of furan-derived BBSOA in the boundary layer (3.5 days under the standard solar spectrum or 7 days in the atmosphere) and lower free troposphere (10 days under the standard solar spectrum or 20 days in the atmosphere), indicating that the mass fraction of SOA derived from 3-methylfuran, 2-methylfuran, and furfural can decrease significantly with photolysis under the solar spectrum (6–23% for 3.5 solar days and 10–40% for 10 solar days). Clearly, there are uncertainties shown as shaded areas in this model. The main source of uncertainty in the model is the uncertainty in cutoff wavelength between two UV bands (see section S1). Other uncertainties include the use of a constant quantum yield within each UV bands. However, we conclude that the direct photolysis of such SOA may be an important fate of SOA under atmospheric conditions, although a large fraction of SOA might be nonphotolabile, consistent with a previous work by O’Brien and Kroll.^56^

It is noted that the study presented here only measured photolysis of SOA under dry conditions (<1% RH) in the QCM cell, though the SOA was produced under both dry and humid conditions. Baboomian et al.^43^ and Wong et al.^60^ measured an increase in mass loss rate by a factor of 2–3 during photolysis on α-pinene SOA at higher RH from laboratory chamber and QCM experiments. The photolytic mass loss results reported for dry conditions here should be interpreted as a lower limit estimate, as the photolysis in the atmosphere that occurred under a higher relative humidity can be faster. Also, the temperature can possibly affect the photolysis of SOA. Photolysis under low-temperature conditions relevant to the upper troposphere conditions should be investigated in future studies. We also noted that the mass evaporation kinetics measured by QCM may not be identical with the photolysis rates of SOA, as the slow evaporation of preexisting and photolytically produced S/IVOC may bias the results. Our measurements indicate that the mass loss during the light-off periods between two light-on periods was faster than dry evaporation, suggesting the formation of S/IVOCs. Nevertheless, previous studies show that photodissociation of SOA mainly produces VOCs, and the measured mass loss rates should be mainly kinetically limited by the photolysis rates. For example, Mang et al.^28^ reported the formation of CO, CH_4_, acetone, and other VOCs via Norrish type I and II reactions during the photolysis of limonene-derived SOA on the filter and in aqueous solutions.^27^ However, different mechanisms may occur for different types of SOA.

In summary, furan compounds have been considered to be an important precursor of brown carbon, especially during biomass burning events, and they have the potential to influence the overall aerosol burden in the atmosphere. As demonstrated in this study, furan SOA can undergo photolysis, leading to a mass reduction through photolytically induced volatilization. We also observed that the mass fraction loss of SOA can vary depending on its chemical characteristics, with strong associations between the mass fraction loss and the nitrogen-containing fraction and light-absorbing species across the different SOA types. These findings suggest that the photolysis of biomass burning precursor-derived SOA under solar irradiance could potentially decrease the OA mass concentration under atmospheric conditions. However, a substantial fraction of SOA mass (60–90%) may remain nonphotolabile during its atmospheric lifetime. Besides, photosensitization triggered by light-absorbing species may be a potential pathway of causing a reduction in the mass, although detailed chemical analysis is needed to further investigate this mechanism. Further research is needed to elucidate the photolytic decomposition pathways of SOA and the impact on the SOA properties. Overall, our findings highlight the range of impact of photolysis on SOA mass from different biomass burning VOC precursors under UV wavelengths relevant to the solar spectrum in tropospheric conditions. Therefore, the results can have a significant implication for climate change projections by improving the representation of organic aerosols in the atmospheric models.

## Data Availability

The chamber experiment data are available online at the Index of Chamber Atmospheric Research in the United States (ICARUS, https://icarus.ucdavis.edu/).

## Supporting Information Available

Texts with corresponding equations used for optimization and atmospheric implication model analysis, tables presenting measured values for each experiment, and figures illustrating the experimental setup, experiment reproducibility, photon flux density of lights, UV–Vis spectra, analyzed furan-derived SOA composition, and comparison of *f*_43_ to mass fraction loss under 300 nm UV light (PDF)
