# Supplementary material for: Photolytic Mass Loss of Secondary Organic Aerosol Derived from Photooxidation of Biomass Burning Furan Precursors
Source: ACS EST Air. 2025 Mar 11;2(4):476–85. doi: 10.1021/acsestair.4c00230 (PMC11997955; doi:10.1021/acsestair.4c00230)
Supplement: Supplementary file 1 — ea4c00230_si_001.pdf [file ea4c00230_si_001.pdf]

Supplementary Information

Photolytic Mass Loss of Secondary Organic Aerosol Derived from Photooxidation of Biomass Burning Furan Precursors

Nara Shin[1], Bin Bai[1], Taekyu Joo[1,2], Yuchen Wang[3,4], Nga L. Ng[1,3,5*], Pengfei Liu[1*]

[1] School of Earth and Atmospheric Sciences, Georgia Institute of Technology, Atlanta, Georgia 30332, United States
[2]Department of Earth and Environmental Sciences, Korea University, Seoul, 02841 South Korea
[3]School of Chemical and Biomolecular Engineering, Georgia Institute of Technology, Atlanta, Georgia 30332, United States
[4]College of Environmental Science and Engineering, Hunan University, Changsha, Hunan 410082, China
[5]School of Civil and Environmental Engineering, Georgia Institute of Technology, Atlanta, Georgia 30332, United States

*Corresponding author: Pengfei Liu (pengfei.liu@eas.gatech.edu), Nga L. Ng (ng@chbe.gatech.edu)

Submitted to: *ES&T Air*

September 2024

Contents of this file
Text S1 and S2
Table S1 to S3
Figure S1 to S4

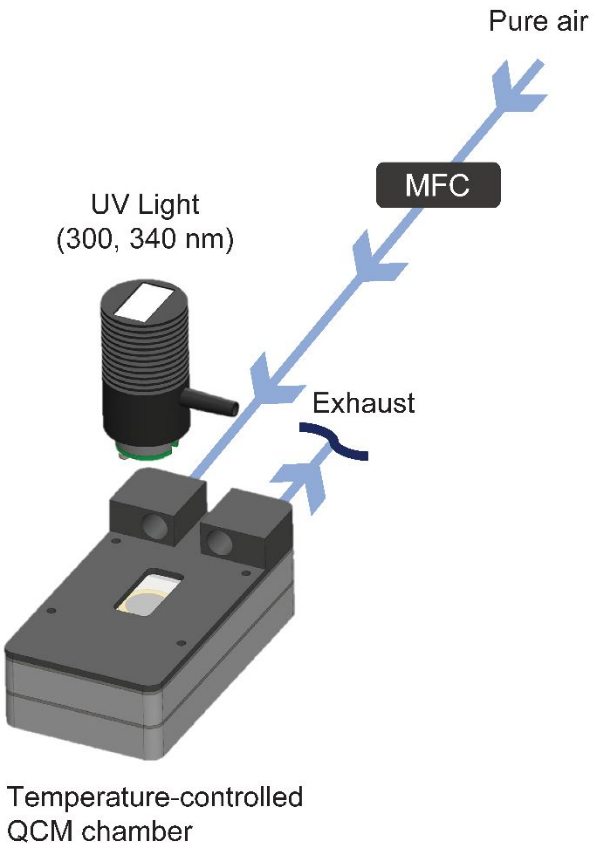

Figure S1. Experiment setup of SOA photolysis using quartz crystal microbalance (QCM).

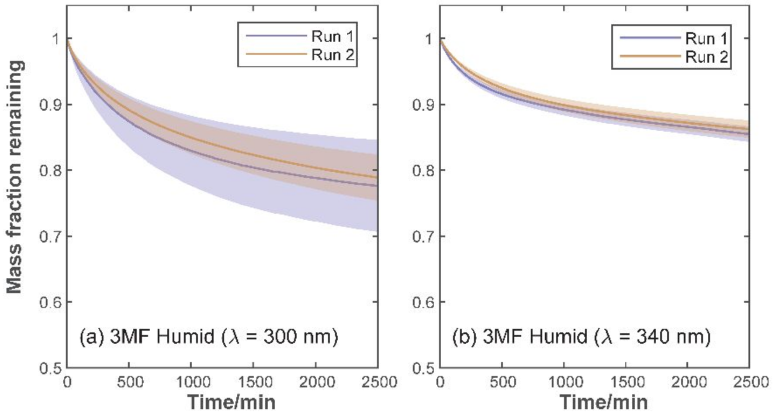

Figure S2. Experimental replications demonstrating the reproducibility of QCM-measured photolytic mass loss. SOA particles were produced from 3-methylfuran under humid conditions in the chamber. Photolysis experiments were conducted under (a) 300 nm and (b) 340 nm UV lights in the QCM flow cell. The sample for Run 1 in panel (a) was collected using the single-

stage impactor, and other samples were collected using the nanometer aerosol sampler. Shaded areas indicate the uncertainties of QCM measurements based on responses in different overtones.

S1. Prediction of mass loss under solar spectrum

In this part of the study, the developed model includes the standard solar spectrum coupled to the flux of lights to simulate the potential impact of photolysis on SOA. A small frequency offset occurs when the UV lamp is turned on. The relationship between lamp frequency offsets (Hz) in the QCM to total irradiance (W/m^2) is obtained by light calibration using a power energy meter (PM100D with S405C sensor, Thorlabs), and the total irradiance of UV lamp (E_1) for each experiment can be therefore accurately determined. We then calculated the spectral photon flux of the lamp $F_{Lamp,\lambda}$ based on the known photon flux spectral distribution $f_{Lamp,\lambda}$ of the UV lamp by the manufacturers' specification, and the total irradiance, as shown in equations S1 and S2:

$$C_{Lamp} = \frac{E_1}{\int \mathrm{f}_{Lamp,\lambda} e_\lambda d\lambda} \quad \text{(S1)}$$

$$F_{Lamp,\lambda} = C_{Lamp} \mathrm{f}_{Lamp,\lambda} \quad \text{(S2)}$$

The calculated $F_{Lamp,\lambda}$ values for two UV lamps in typical experiments are shown in Figure S2. As a comparison, a standard solar irradiance spectrum (ASTM G-173-03, National Renewable Energy Laboratory) is converted into photon flux using Planck-Einstein relation using Eqs. S3 and S4 (Figure S2):

$$e_\lambda = h\frac{c}{\lambda} \quad \text{(S3)}$$

$$F_\lambda = \frac{E_\lambda}{e_\lambda} \quad \text{(S4)}$$

where h is Plank's constant ($6.626 \times 10^{-34}\ J/s$), c is a speed of light ($3.00 \times 10^8\ m/s$), λ is a wavelength (*nm*), e is photon energy (*J/photon*), E is irradiance spectrum ($W \cdot m^{-2} \cdot nm^{-1}$), and F is photon flux ($Photon \cdot cm^{-2} \cdot s^{-1} \cdot nm^{-1}$).

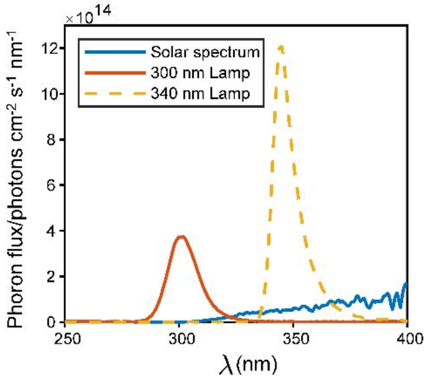

Figure S3. Spectral photon flux density of 300 nm light, 340 nm light, and solar spectrum (ASTM G-173-03).

With the wavelength-dependent absorption cross-section, the photolysis rate constant of solar radiation (j_{solar}) is also calculated based on equation S5. The ratio between j_{lamp} and j_{solar} is

used to derive the scaling factors of each lamp. With the application of scaling factors, the mass fraction loss of each experiment is regenerated equivalent to solar radiation. The scaling factors of photolysis rates between lamps and the solar radiation could be calculated using the following equations S5-9:

$$J = \int F_\lambda \Phi_\lambda \sigma_\lambda d\lambda \quad \text{(S5)}$$

$$J_{\text{L 1}} = \int F_{L1,\lambda}\sigma_\lambda \Phi_{L1,\lambda} d\lambda = \Phi_{L1} \int F_{L1,\lambda}\sigma_\lambda d\lambda \quad \text{(S6)}$$

$$J_{\text{L 2}} = \int F_{L2,\lambda}\sigma_\lambda \Phi_{L2,\lambda} d\lambda = \Phi_{L2} \int F_{L2,\lambda}\sigma_\lambda d\lambda \quad \text{(S7)}$$

$$J_{\text{solar} <\lambda_{cut}} = \int_{280\ nm}^{\lambda_{cut}} F_{solar,\lambda}\sigma_\lambda \Phi_{solar,\lambda} d\lambda = \Phi_{solar<\lambda_{cut}} \int_{280\ nm}^{\lambda_{cut}} F_{solar,\lambda}\sigma_\lambda d\lambda \quad \text{(S8)}$$

$$J_{\text{solar} >\lambda_{cut}} = \int_{\lambda_{cut}}^{400\ nm} F_{solar,\lambda}\sigma_\lambda \Phi_{solar,\lambda} d\lambda = \Phi_{solar>\lambda_{cut}} \int_{\lambda_{cut}}^{400\ nm} F_{solar,\lambda}\sigma_\lambda d\lambda \quad \text{(S9)}$$

Where F_L is the photon flux of the lamp, σ_λ is the absorption cross-section, and $\Phi_{L,\lambda}$, quantum yields of lamps are assumed to be constant within the wavelength range of each lamp. The absorbance at a specific wavelength can be calculated using the following equation S10:

$$\sigma_\lambda = \log(10)\frac{Abs_\lambda}{NL} = \log(10)\frac{Abs_\lambda}{\rho L} MW_{SOA} \quad \text{(S10)}$$

Abs_λ is the absorbance measured by UV-Vis spectrometry measurement (DT-Mini and USB4000, Ocean Optics) as a function of wavelength from the previous study[1], N is the molecule concentration of SOA in the extract, ρ is the mass concentration of the extraction solution, and L is the optical depth (2.5 *m*) used during UV-Vis spectrometer measurement. In this study, the choice of molecular weight of the SOA was assumed to be 200 g/mol, based on our previous FIGAERO-HR-ToF-CIMS measurements.[1]

The derivation of the scaling factor from lamps to 2 ranges of the solar spectrum can be expressed as R_L (equations S11-12):

$$R_{L1} = \frac{J_{\text{solar} <\lambda_{cut}}}{J_{\text{L1}}} = \frac{\int F_{L1,\lambda}\sigma_\lambda\, d\lambda}{\int_{280\ nm}^{\lambda_{cut}} F_{solar,\lambda}\sigma_\lambda\, d\lambda} \quad \text{(S11)}$$

$$R_{L2} = \frac{J_{\text{solar} >\lambda_{cut}}}{J_{\text{L2}}} = \frac{\int F_{L2,\lambda}\sigma_\lambda\, d\lambda}{\int_{\lambda_{cut}}^{400\ nm} F_{solar,\lambda}\sigma_\lambda\, d\lambda} \quad \text{(S12)}$$

The decay rates of the photolysis of furan-derived SOA were calculated by equations S13-16.

$$m_{L1} = A_0 + A_1\exp(-k_1 t) \quad \text{(S13)}$$

$$m_{L2} = B_0 + B_1\exp(-\mathrm{g}_1 t) \quad \text{(S14)}$$

If, $A_0 < B_0$

$$f_1 = A_0; f_2 = (B_0 - A_0)\exp(-k_1 R_{L1} t); f_3 = (1 - B_0)\exp(-R_{L1}k_1 t - R_{L2}\mathrm{g}_1 t)\ ) \quad \text{(S15)}$$

If, $B_0 < A_0$

$$f_1 = B_0; f_2 = (A_0 - B_0)\exp(-g_1 R_{L2} t); f_3 = (1 - A_0)\exp(-R_{L1}k_1 t - R_{L2}\mathrm{g}_1 t) \quad \text{(S16)}$$

To fit the experimental data, we used an exponential model, A_0 and B_0 represent the fraction of the un-photolyzable SOA, A_1 and B_1 as photolyzable fraction of SOA with decay rate of k_1 and g_1, respectively for each lamp. The summation of the components $A_0 + A_1$ and $B_0 + B_1$ were normalized to 1 and the photolysis curve under the solar radiation could be generated by equation S17.

$$m_{solar} = f_1 + f_2 + f_3 \quad \text{(S17)}$$

Table S1. Experimental conditions measured by GC-FID, HR-ToF-AMS, SMPS, and QCM.

Experiment	3-methylfuran SOA		2-methylfuran SOA		Furfural SOA	
RH condition in chamber	Dry (<5%)	Humid (50-55%)	Dry (<5%)	Humid (50-55%)	Dry (<5%)	Humid (50-55%)
ΔHC (ppb)	520.67 ± 1.94	533.58 ± 2.65	614.63 ± 1.87	603.44 ±4.90	197.49 ±3.45	201.64 ± 3.28
Elemental ratio (H:C, O:C)	1.56, 1.24	1.54, 1.20	1.39, 1.49	1.41, 1.55	1.15, 1.38	1.20, 0.94
Mass on QCM sensor (μg) collected by impactor	17.2, 9.4, 10.0		5.9, 5.2, 9.4	11.7, 4.9	9.3, 8.8, 14.8	
Mass on QCM sensor (μg) collected by precipitator		4.3, 3.6, 5.3		13.7,		4.0, 5.5, 4.9

Table S2. Final organic mass fraction loss under different conditions over 48 hours

	SOA precursor	Dark (%)	300 nm (%)	340 nm (%)
Dry	3-Methtlfuran	2.7	24.5	8.9
	2-Methtlfuran	5.1	26.2	11.9
	Furfural	3.9	35.8	6.8
Humid	3-Methtlfuran	4.2	22.0	14.9
	2-Methtlfuran	3.6	16.5	7.0
	Furfural	2.6	23.9	10.2

S2. Measurements of mass absorption efficiency (MAE)

In this part of the study, organic aerosol samples were collected on 47 mm Teflon filters (2 μm pore size, Pall Corporation) during the peak aerosol mass concentration. The collected organic aerosol was dissolved in methanol and water to extract soluble organic aerosol. However, in this specific study, we only used the absorbance of organic aerosol dissolved in a methanol solution,

which was close to the total organic aerosol mass deposited on the filter samples. Detailed descriptions of filter collection were described in Joo et al. (2024)[1]. The mass absorption efficiency (MAE) at 310 nm is estimated based on the measured absorbance (abs_{λ}) followed by equation S18 for each compound from the UV-Vis spectrometer described previously. The absorbance abs_{λ} was measured for the wavelength range between 305 to 315 nm followed by smoothing a high noise curve to obtain $MAE_{310\ nm}$.

$$MAE_{310\ nm}\left(m^2/g\right) = \frac{\frac{\sum_{305\ nm}^{315\ nm} abs_{\lambda}}{11}}{Total\ soluable\ organic\ aerosol\ (\ g\ \ m^{-2})} \qquad \text{(S18)}$$

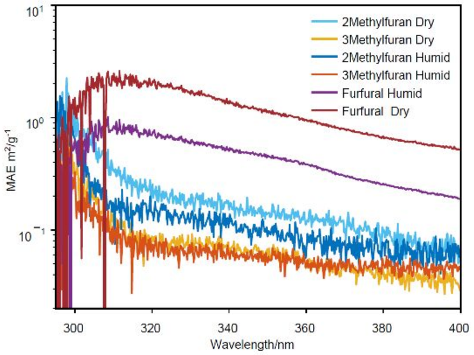

Figure S4. UV-Vis spectra analysis of SOA derived from biomass burning precursors.

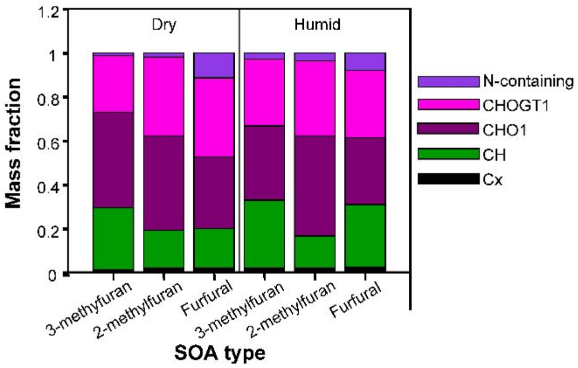

Figure S5. Typical composition of furan SOA derived measured by the HR-ToF-AMS. It is noted that the NO family is not included in the calculation of the mass fraction. CHOGT1: CxHyOz family where x>=1, y>=0, z>1; CHO1: CxHyOz family where x>=1, y>=0, z=1; CH: CxHy family where x>=1 and y>=1; Cx: Cx family where x>=1.

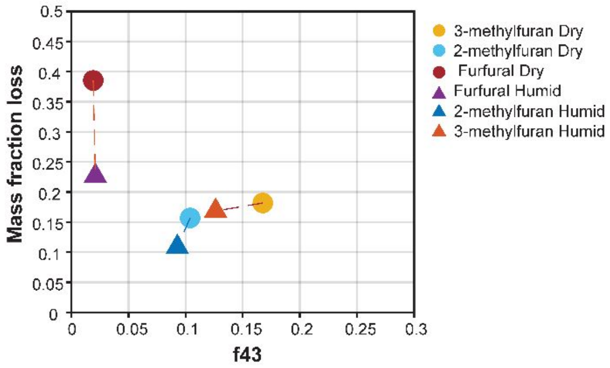

Figure S6. Comparison of f43 to mass fraction loss after 48-hour photolysis under 300 nm UV light.

Table S3. Summary of relative reduction (%) of BBSOA mass due to photolysis under solar spectrum.

	SOA precursor	24 hr.	48 hr.	72 hr.	84 hr. (7 days in ambient)	120 hr.	240 hr. (20 days in ambient)
Dry	3-methylfuran	6.1%	9.1%	11.0%	11.8%	13.7%	18.2%
	2-methylfuran	5.0%	7.9%	9.8%	10.5%	12.1%	15.7%
	Furfural	8.5%	15.2%	20.6%	22.9%	28.5%	38.5%
Humid	3-methylfuran	8.7%	11.8%	13.3%	13.8%	14.8%	16.8%
	2-methylfuran	2.5%	4.6%	6.2%	6.7%	8.2%	10.8%
	Furfural	8.6%	13.6%	16.7%	17.9%	20.1%	22.6%

References

(1) Joo, T.; Machesky, J. E.; Zeng, L.; Hass-Mitchell, T.; Weber, R. J.; Gentner, D. R.; Ng, N. L. Secondary Brown Carbon Formation From Photooxidation of Furans From Biomass Burning. *Geophys Res Lett* **2024**, *51* (1). https://doi.org/10.1029/2023GL104900.
